# Prevalence and causes of social media usage and addiction status of pregnant women

**DOI:** 10.1186/s12905-023-02787-1

**Published:** 2023-12-08

**Authors:** Merve Bağrici Bozan, Eda Cangöl

**Affiliations:** 1 Independent Researcher, Çanakkale, Turkey; 2 Department of Midwifery, Faculty of Health Sciences, Çanakkale Onsekiz Mart University, Çanakkale, Turkey

**Keywords:** Pregnancy, Prevalence, Social media, Social media addiction

## Abstract

**Background:**

This study was conducted to determine the prevalence and causes of social media usage and the addiction status of pregnant.

**Materials and methods:**

This cross-sectional study, which was conducted in the Gynecology and Obstetrics Clinics of a university and state hospital between December 2019 and February 2020 in a city in the west of Turkey, aims to determine the prevalence and causes of social media usage and the addiction status of pregnant women. 500 people were included in the study. The data were collected with the face-to-face interview technique in the outpatient clinics.

**Results:**

The prevalence of the pregnant women’s social media usage was found to be 100%. The average daily social media usage was 3.5 h, whereas the social media addiction index was X̅ = 65.58. There were 358 pregnant women (71.6%) with high social media addiction scores.

**Conclusions:**

In the study, it was concluded that all the pregnant women used social media and that most of them had a high level of social media addiction. Healthcare professionals should promote the conscious and balanced use of social media by organizing educational programs related to the accessibility of accurate and reliable online content for pregnant women at every stage of healthcare, and the dangers and addiction of social media.

## Introduction

As the internet continues to penetrate the lives of an increasing number of individuals, it has established itself as an indispensable tool for communication. The wide range of uses for the internet, from leisure activities to professional purposes, as well as its ability to meet various needs from communication to daily work and academics, has only further solidified its central role in our lives [1]. According to the Global Digital 2019 report, 45% of the population in the world uses social media [2]. In Turkey, according to the Household ICT Usage Survey of the Turkey Statistical Institute, the rate of internet use among individuals in the 16–74 age group in August 2020 was found to be 79%. Online communities are practical tools for obtaining information on needed topics. In the study of Hadımlı et al. [3], 58.2% of 134 pregnant women stated that they used the internet for 21 h or more in a week. In the other study, most of the pregnant women followed social media pages about their pregnancies and conducted research on their pregnancies on the internet [4]. While healthcare providers are deemed the most trustworthy source of information for expectant mothers, a majority of them turn to social media to quickly access information [4, 5].

Apart from the physical health of pregnant women, the risk of mental health in terms of social media addiction should be evaluated. Psychological disorders such as stress [6], anxiety [7], sleep disorders [8], and depression [9] are also observed as a consequence of social media addiction. While individuals, who spend a long time on social media platforms, expose themselves to the perfectionist profiles of others heavily, the assumption that those people have happier and more successful lives is constantly reinforced, which results in social isolation and increases the susceptibility to depression [10, 11]. When pregnant women refer to social media for information on various pregnancy-related issues [12], including nutrition, birth and infant development, the accuracy of the information they obtain is often not proven, causing women to be misinformed and unnecessarily worried about pregnancy-related issues and mental health issues and leading them to feel inadequate and stressed. Studies on the use of social media by pregnant women are extremely limited in the literature. No studies were found on social media addiction of pregnant women. In this context, this study was conducted to examine the prevalence, reasons and addiction status of pregnant women women using social media.

**Research questions**.

1. What is the prevalence of pregnant women using social media?
2. Why do pregnant women use social media?
3. What is the social media addiction rate of pregnant women?

## Method

### Design, data collection and sample

This cross-sectional study was conducted with pregnant women who applied to the Obstetrics and Gynecology Clinics of a university and state hospital between December 2019 and February 2020 in a city in the west of Turkey. The unknown universe sampling method was used to determine the number of people to be sampled in the research. In the sample calculation, the formula n=${t}^{2 }pq/{d}^{2}$, where the population is unknown, was used [13]. The minimum sample size to be reached was found to be 323. However, considering the possible losses in the research, the sample was completed with 500 people.

**Inclusion criteria in the study**

- speaks Turkish.
- literate.
- Pregnant women willing to participate in the study.
- Pregnant women over the age of 18 with a civil marriage.

**Exclusion criteria for the study**

- Those who were diagnosed with any psychological disorder were excluded from the study.

### Data collection methods

#### Data collection form

The data collection form consists of two parts. Prepared by the researchers by scanning the literature [4, 14–16]. The first part consists of 9 questions questioning the socio-demographic and obstetric characteristics of the pregnant women participating in the study. The second part includes questions about social media usage during pregnancy and consists of 15 questions.

#### Social Media Addiction Scale-Adult Form (SMAS-AF)

The Şahin and Yağcı [15] developed a scale, named SMAS-AF, for measuring social media addiction levels in adults aged 18 to 65. Validity studies were conducted and both explanatory and confirmatory analyses were performed. It was established that the SMAS-AF scale has a five-point Likert format, two sub-components (virtual tolerance and virtual communication), and 20 items. Virtual tolerance sub-dimension consists of items 1–11, and virtual communication consists of items 12–20. Items 5 and 11 are reverse scored. The highest score that can be obtained from the scale is 100 and the lowest score is 20. A high score is considered to mean that the individual perceives himself as a “social media addict”. The Cronbach Alpha internal consistency coefficient was 0.94 for the overall scale, 0.92 for virtual tolerance, and 0.91 for virtual communication. In this study, the Cronbach’s Alpha number for the overall scale was determined as 0.907.

#### Data collection

The data was collected by the researcher through face-to-face interviews with pregnant women who agreed to participate in the study between December 2019 and February 2020. Participation is on a voluntary basis. A consent form developed to provide information about confidentiality and research purposes has been added. The overall interview lasted approximately 20 min.

### Data analysis

The frequency analysis findings were included in order to reveal the socio-demographic characteristics and the social media usage status of the participants. The Skewness and Kurtosis values ​​obtained for the Social Media Addiction (Skewness: 0.467; Kurtosis: 0.099) scale used in the study were between − 2.0 and + 2.0; therefore, it is noteworthy that the data showed a normal distribution [17]. Based on these results, IBM SPSS 21.0 program was used for the analysis of the data. While evaluating the study data, in addition to descriptive statistical methods (mean, standard deviation, median, frequency, minimum, maximum), the Independent Samples T-Test was used for comparing normally distributed variables in two groups, whereas the Mann-Whitney U test was used for two group comparisons of variables that did not show normal distribution. The One-Way ANOVA test was used in the comparison of three and more normally distributed groups, while the Tukey-Dunnett’s C post hoc tests were used to identify the groups that caused the difference. The Kruskal Wallis H test was used for comparisons of three and more groups that did not show normal distribution, and the Mann-Whitney U test was used to identify the groups that caused the difference. In statistical analysis, significance was evaluated at the p < 0.05 level.

## Results

Table 1 examines the findings regarding the socio-demographic and obstetric characteristics of the participants. The average age of the participants is 27.6 ± 5.837 and the median age is 26 (Min: 18 Max: 44). Approximately 70.6% of the participants are under 30 years old (Table 1).

**Table 1:** Distribution of data regarding socio-demographic and obstetric characteristics of participants (n = 500)

Characteristic	Number (n)	%
**Age**		
18–24	161	32,2
25–34	270	54,0
35 and above	69	13,8
**Employment Status**		
Employed	177	35,4
Unemployed	323	64,6
**Income Status**		
Low	96	19,2
Moderate	219	43,8
Good	160	32,0
Very good	25	5,0
**Educational Status**		
Literate	24	4,8
Elementary school	78	15,6
High School	169	33,8
Bachelor’s degree and higher	229	45,8
**Husband’s educational status**		
Literate	29	5,8
Elementary school	198	39,6
High School	153	30,6
Bachelor’s degree and higher	120	24,0
**Personel habits**		
None 449 89,8 Smoking 15 3 Alcohol 36 7,2 **Number of Pregnancy**		
1 Pregnancy 2 Pregnancies	107	21,4
	127	25,4
3 Pregnancies	172	34,4
4 Pregnancies and more	94	18,8
**Number of Births**		
None	110	22,0
1 Births	183	36,6
2 Births	142	28,4
3 Births and more	65	13,0
**Attendance to pregnancy trainings/classes**		
Yes	220	44,0
No	280	56,0

In Tables 2, the findings regarding the social media usage situation of the participants are examined. It is seen that the way participants connect to these platforms is usually by mobile phone or tablet (n = 273), and 42.4% (n = 212) connect to social media by both a computer and a mobile phone/tablet. It was found that 483 pregnant women (96.6%) rarely used social media before their pregnancy and that the rate of those who did not use social media before pregnancy was 3.4% (n = 17) (Table 2). The pregnant women gave more than one reason for using social media. Of these reasons, 228 (45.6%) of the participants stated that they used social media for social networking when they were asked the question ‘Which one of them pushes you to use social media?‘. It was determined that the most common reason for using social media after social networking was about obtaining information with 20.2%.

**Table 2:** Findings regarding the social media usage of participants

Characteristic	Number (n)	%
**Social Media Usage Before Pregnancy**		
Never	17	3,4
Rarely	483	96,6
**The Way Participants Connect to Social Media**		
Computer	15	3,0
Mobile Phone/Tablet	273	54,6
Both	212	42,4
**Purpose of Using Social Media**		
Social networking	228	45,6
Contact	88	17,6
Shopping	61	12,2
Obtaining information	101	20,2
Research	22	4,4
**Sources of Blogs and Articles on Pregnancy and Baby Followed on Social Media**		
Healthcare Professional Blogs and Pages	108	21,6
Doula	50	10,0
Blogger Mothers	273	54,6
Mom and Baby Brands	69	13,8
**The First Person that Participants Contact on Social Media on Any Matters Related to Pregnancy and Baby**		
Healthcare Professional Blogs and Pages	168	33,6
Doula	62	12,4
Blogger Mothers	231	46,2
Mom and Baby Brands	39	7,8
**Status of Believing the Accuracy of Information on Social Media**		
I sometimes believe	133	26,6
I often believe	199	39,8
I always believe	168	33,6
**Status of Participants regarding whether Information Researched by Participants on Social Media Affects Their Decision**		
Yes	403	80,6
No	97	19,4
**Status of Participants regarding Consulting Health Professionals about Information on Social Media**		
Yes	236	47,2
No	264	52,8
**Status of Participants regarding Doing Research on a Pregnancy and Baby Product before buying it**		
Yes	392	78,4
No	108	21,6

In the study, it was determined that the average daily social media usage of the participants was 3.536 ± 1.3916 h (min: 1 h; max: 8 h). 78% of the pregnant women (n = 390) stated that their daily social media usage time was 3 h or more. Looking at the social media platforms used by the participants, it was found that the application of Instagram was the mostly used one with 421 pregnant women (84.2%). The other most used social media platform was Facebook, used by 74.4% (n = 372) of the participants.

The pages followed by the participants on social media to do research and obtain information are blogger mothers with 54.6% (n = 273). Again, it was determined that the first source that the participants referred to on social media on any issue related to pregnancy and baby was blogger mothers with 46.2% (n = 231). Healthcare professionals (33.6%) (n = 168) are the second group that the participant consulted on social media as a source of access to information. All of the participants chose one of the options ‘I sometimes believe’ (26.6%), ‘I often believe’ (39.8%) and ‘I always believe’ (33.6%) about the information they encounter on social media. No pregnant woman was found who ignored any information she encountered on social media (Table 2).

It was investigated why the participants met their information needs about their pregnancy from social media. The pregnant women were given the right to answer these reasons more than once. Most of the participants preferred to obtain information from social media due to reasons such as accessing the desired information immediately (73.8%, n = 369) and difficulty in reaching health professionals (64.6%, n = 323). 42% (n = 211) of the participants stated that they did research on social media because they were reluctant to ask information about their pregnancy to a healthcare professional or a relative who had a similar situation. In Table 3, the comparison of the social media addiction scale total score averages with some variables was examined. There was a significant difference between the social media addiction of the pregnant women and their income status, educational status, educational status of their husbands, number of pregnancies, number of births, and participation in pregnancy trainings (p < 0.05). At this point, the social media addiction level of the pregnant women with low income and low educational status who had many children and a husband with a low educational status and did not attend a pregnancy education was higher. In addition, it was seen that using social media for long hours, frequently using social media before pregnancy, using social media mostly for social networking, and generally following blogger mothers on social media affected social media addiction. Again, in terms of social media addiction, a significant difference was found between those who were the first to refer to bloggers on social media and those who always believed in the information on social media (p < 0.05). In terms of social media addiction, a statistically significant difference was found between those who believed in information obtained from social media and those who did not consult health professionals (p < 0.05) (Table 3).

**Table 3:** Comparison of social media addiction scale total score averages with some variables

	X ± SS	*F *X^2^ *t	p	**
**Income Status** Low Moderate Good Very good	70,24 ± 13,81 65,18 ± 13,89 64,16 ± 13,13 60,24 ± 18,83	f: 5,432	*0,001	Low-Moderate, Low-Good, Low-Very Good
**Educational Status** Literate Elementary School Secondary School Bachelor’s degree and higher	68,58 ± 17,30 69,27 ± 14,69 65,00 ± 13,60 64,42 ± 13,74	f: 2,778	*0,041	Literate- Bachelor’s degree and higher
**Husband’s educational status** Literate Elementary School Secondary School Bachelor’s degree and higher	68,79 ± 16,02 68,63 ± 12,26 64,46 ± 13,39 61,18 ± 16,02	*: 8,149	*0,000	Literate- Bachelor’s degree and higher, Elementary School- Secondary School, Elementary School- Bachelor’s degree and higher
**Number of Pregnancy** 1 pregnancy 2 pregnancies 3 pregnancies 4 pregnancies and more	59,08 ± 16,09 65,87 ± 13,60 67,34 ± 12,71 69,35 ± 12,48	X^2^: 28,725	*0,000	1 pregnancy-2 pregnancies, 1pregnancy - 3pregnancies, 1pregnancy - 4pregnancies and more
**Number of Births** None 1 birth 2 births 3 births and more	62,61 ± 15,59 65,59 ± 14,24 65,93 ± 13,08 69,80 ± 12,22	X^2^ : 8,876	*0,031	None-3 births and more, 2 births-3 births and more
**Attendance to Pregnancy Trainings** Yes No	64,06 ± 14,19 66,77 ± 13,95	t: -2,140	*0,033	
**Social Media Usage Prevalence** 0–3 h 3–6 h 6 h and more	54,03 ± 16,38 68,47 ± 10,94 71,88 ± 15,00	f: 59,782	*0,000	0–3 h-(3–6) hours, 0–3-6 h and more
**Social Media Use Before Pregnancy** Never Rarely Often	61,12 ± 13,24 57,77 ± 15,68 68,28 ± 12,58	f: 28,284	*0,000	Rarely-Often
**Purpose of Using Social Media** Social networking Contact Shopping Obtaining information Research	69,73 ± 12,44 64,23 ± 12,57 64,57 ± 13,61 60,37 ± 14,99 54,64 ± 18,57	X^2^ : 42,713	*0,000	Social networking- Contact, Social networking- Shopping, Social networking- Obtaining information, Social networking- Research, Contact- Research, Shopping- Research
**Blogs and Other Written Sources Followed** Healthcare Professionals Doula Blogger Mothers Mom and Baby Brands	60,61 ± 17,47 65,84 ± 11,04 68,27 ± 12,05 62,51 ± 15,25	X^2^ : 20,706	*0,000	Healthcare Professionals- Blogger Mothers, Blogger Mothers- Mom and Baby Brands
**The First Person that Participants Contact** Healthcare Professionals Doula Blogger Mothers Mom and Baby Brands	61,37 ± 16,41 65,79 ± 12,65 68,81 ± 11,73 64,18 ± 13,96	f: 9,651	*0,000	Healthcare Professionals- Blogger Mothers
**Status of Believing the Accuracy of Information** Sometimes Often Always	56,81 ± 15,50 68,75 ± 12,92 68,75 ± 11,11	X^2^ : 69,061	*0,000	Sometimes-Often, Sometimes-Always
**Status of affecting the Decision Process** Yes No	66,56 ± 13,98 61,49 ± 13,95	t: 3,212	*0,001	
**Consulting Health Professionals** Yes No	62,13 ± 15,39 68,66 ± 12,07	t:-5,232	*0,000	

*= p < 0.05

**=Demonstrate significant difference between which groups

*F Anova Test Score

*t Independent Samples t Test Score

*Chi-Square Test Score for X^2^ Test

When the statements of the participants about the use of social media are examined, it was concluded that the pregnant women agreed more with the following judgments: “Upon waking up, my first task of the day is to log onto social media.” (3.26%), “I feel the need to constantly be on social media in order to stay informed about current events.” (3.56%), “I find my online friendships on social media to be more desirable than my in-person relationships” (3.15%), and " Even when in the company of others, I choose to spend my time on social media rather than engaging with them” (3.19%).

## Discussion

It is noteworthy that when the points given by the pregnant women to the items in the SMAS-AF are added together, the lowest point was 24 and the highest point was 97. The average social media addiction level of the pregnant women was X̅ = 65.58. When we look at the social media score distribution of the pregnant women outside of the general average, it was found that there were 358 pregnant women (71.6%) between 61 and 100 points. According to Şahin & Yağcı [15], who conducted the validity and reliability study of the Social Media Addiction Scale-Adult Form, as the average scores of the participants increased, the level of addiction also increased. According to the results of the study conducted in this context, it can be stated that approximately 7 out of 10 pregnant women have problems with social media addiction. The high rate of being online for long hours on social media also causes some health problems. It is thought that the use of mobile devices creates oxidative stress and may increase the risk of cancer in this way [18]. In addition, there are psychological problems caused by social media addiction.

Excessive or problematic use of the internet and social media and spending at least 8.5 to 21.5 hours online a week [19] are considered as addiction. In this study, the average daily social media usage of the pregnant women was found to be 3.5 hours. 78% of the pregnant women used daily social media for 3 hours or more. The prevalence of the social media usage of the pregnant women was determined as 100%. In addition, 96’6% (n = 483) of the pregnant women stated that they used social media before their pregnancy. A significant difference was found between the pregnant women who used social media before pregnancy and those who did not use or rarely used it. In the study of Öztürk and Uzunkaya [20], the prevalence of internet use among pregnant women was 70.6%. The results of the study on the usage of social media by pregnant women align with the findings of previous research and demonstrate a high rate of utilization. In the study by Wallwiener et al. [21], the pregnant women reported an average of 2.38 hours per week spent on websites related to pregnancy and 3.41 hours using mobile apps. The ‘We are social’ report indicates that individuals between the ages of 16–64 spend an average of 2 h and 51 min daily on social media. Globally, the average time spent on social media by users is 2 h and 24 min per day [22]. According to this result, our country spends a longer period of time than the world in terms of social media usage. The use of social media for long hours directly affects social media addiction. In this study, a significant difference was found between the duration of the social media usage of the pregnant women and their SMAS-AF scores. Considering these reasons, it is thought that the prevalence and addiction status of pregnant women and the accuracy of the information they obtain are extremely important for the continuity of pregnancy and the health of future babies. There is no study in the literature on the social media addiction of pregnant women. In support of the studies carried out, the pregnant women with high SMAS-AF scores stated that they agreed with the following statements: “I have neglected family members because of social media”, “I prefer friendships on social media to real-life friendships.“ and “Even when there is someone around, I prefer to be on social media.“

In this study, it was determined that the pregnant women who did not attend pregnancy trainings had a higher social media addiction level than the pregnant women who attended pregnancy trainings and received education from health professionals. This result is related to the fact that the pregnant women who did not attend pregnancy trainings attempted to meet their need for information via social media. Although health professionals are the most reliable source of information for pregnant women, most of the pregnant women who cannot reach healthcare professionals use the internet to access information immediately [3–5]. A research study found that pregnant women expressed disappointment with the information given by medical staff, as they felt they did not have sufficient time to ask questions. Consequently, they sought information on the internet, with blogs and websites being the most frequently used resources at 85.8% [23]. Another study reported that expectant mothers considered health information found on the internet to be trustworthy and beneficial [12]. The study results are similar. In pregnancy trainings, it is expected that the rate of social media usage will decrease due to the fact that pregnant women, who have received information from healthcare professionals and find answers to their questions, focus on their pregnancy and their future babies.

The reasons for pregnant women to use social media differ from each other. In this study, the reasons for pregnant women to use social media can be listed as follows: social networking, contact, obtaining information, shopping, research. However, the most popular reasons for using it are social networking, obtaining information, contact, shopping and research. It can be said that social network actions, such as sharing photos and articles, interacting with different social media groups about a common purpose, and leaving comments and likes on the content created by another user, are extremely popular for pregnant women. The fact that individuals can reach, interact with and receive feedback from artists, bureaucrats and popular people, who cannot be reached in real life, via social media reveals that communication barriers have been removed. The excessive use of social media has caused a state of addiction. Researchers point out that the desire to enter social media is above the desire to sleep and rest, and this is a social disaster [19]. For example, according to a study conducted by the University of Gothenburg, it was found that 34% of the women between the ages of 18–34 checked their social media accounts even before they went to the toilet when they woke up in the morning and that 39% identified themselves as Facebook addicts [24].

Although there is no significant relationship between gender and social media addiction in other studies in the literature, it was revealed that women engaged in social media more than men, experienced loss of control, used social media to forget their personal problems, relieved their loneliness, and got rid of negative thoughts in their lives [25, 26]. In this context, the fact that women forget everything while using social media shows that social media is a kind of psychological and emotional support tool for them [25]. To the best of our knowledge, there is no study in the literature on the social media addiction of pregnant women. In the study conducted by Ünal and Deniz [26] on university students, it was stated that women received more emotional support from social media than men.

## Conclusion

The study’s findings indicated that all of the pregnant women used social media and a majority had a high level of social media dependence. The results revealed a significant correlation between the frequency of social media usage and the length of time spent on it, as well as addiction to social media. Health literacy should be promoted in all stages of healthcare, especially in primary healthcare services and prenatal schools, by midwives and obstetrics nurses, public health nurses, and psychiatric nurses. They should raise awareness for pregnant women to access current and accurate information on social media platforms. It is necessary to expand efforts and increase collaboration in this regard. Training programs should be organized on the dangers of social media and social media addiction. These recommendations can play a significant role in promoting healthy social media use and in the conscious battle against social media addiction among pregnant women in the broader community.

## Data Availability

The data that support the findings of this study are available from Eda Cangöl but restrictions apply to the availability of these data, which were used under license for the current study, and so are not publicly available. Data are how ever available from the authors upon reasonable request and with permission of Eda Cangöl.
